# Neutralizing Antibodies Induced by Recombinant Virus-Like Particles of Enterovirus 71 Genotype C4 Inhibit Infection at Pre- and Post-attachment Steps

**DOI:** 10.1371/journal.pone.0057601

**Published:** 2013-02-22

**Authors:** Zhiqiang Ku, Xiaohua Ye, Xulin Huang, Yicun Cai, Qingwei Liu, Yan Li, Zhiguo Su, Zhong Huang

**Affiliations:** 1 Key Laboratory of Molecular Virology & Immunology, Institut Pasteur of Shanghai, Shanghai Institutes for Biological Sciences, Chinese Academy of Sciences, Shanghai, China; 2 National Key Laboratory of Biochemical Engineering, Institute of Process Engineering, Chinese Academy of Sciences, Beijing, China; University of Rochester, United States of America

## Abstract

**Background:**

Enterovirus 71 (EV71) is a major causative agent of hand, foot and mouth disease, which has been prevalent in Asia–Pacific regions, causing significant morbidity and mortality in young children. Antibodies elicited by experimental EV71 vaccines could neutralize infection *in vitro* and passively protect animal models from lethal challenge, indicating that neutralizing antibodies play an essential role in protection. However, how neutralizing antibodies inhibit infection *in vitro* remains unclear.

**Methods/Findings:**

In the present study, we explored the mechanisms of neutralization by antibodies against EV71 virus-like particles (VLPs). Recombinant VLPs of EV71 genotype C4 were produced in insect cells using baculovirus vectors. Immunization with the VLPs elicited a high-titer, EV71-specific antibody response in mice. Anti-VLP mouse sera potently neutralized EV71 infection *in vitro*. The neutralizing antibodies in the anti-VLP mouse sera were found to target mainly an extremely conserved epitope (FGEHKQEKDLEYGAC) located at the GH loop of the VP1 protein. The neutralizing anti-VLP antisera were able to inhibit virus binding to target cells efficiently. In addition, post-attachment treatment of virus-bound cells with the anti-VLP antisera also neutralized virus infection, although the antibody concentration required was higher than that of the pre-attachment treatment.

**Conclusions:**

Collectively, our findings represent a valuable addition to the understanding of mechanisms of EV71 neutralization and have strong implications for EV71 vaccine development.

## Introduction

Enterovirus type 71 (EV71) is a member of the enterovirus genus of the picornaviridae family. It possesses a single-stranded, positive-sense RNA genome of ∼7.4 kb, which is packaged in an icosahedral capsid. The viral genome is divided into structural region P1, and non-structural regions P2 and P3 (reviewed in [1]). The P1 translation product can be processed by viral protease 3CD to produce capsid subunit proteins VP1, VP0 and VP3; all three of which co-assemble to form empty viral capsids [2]. A portion of VP0 can be autocleaved to yield VP2 and VP4, which are associated with infectious EV71 virions [2].

EV71 is the major causative agent of hand, foot and mouth disease (HFMD), which frequently occurs in children under the age of 5 years [3], [4]. Over the past several years, there have been many large EV71 outbreaks in the regions and countries of the far East, such as China [5], [6], [7], [8], Vietnam [9], and Korea [10], causing significant morbidity and mortality. Individuals infected with EV71 usually show mild symptoms, such as sores and blisters on the surface of the hand, foot and buttocks, and fever. A portion of the EV71-infected patients may develop severe neurological complications, including polio-like paralysis, brainstem encephalitis, and pulmonary edema, which may ultimately result in death [1], [3], [4]. However, no specific vaccine is yet available to prevent EV71 infection. EV71 has been classified into three genogroups (A, B and C), which can be further divided into 11 genotypes (A, B1–B5, and C1–C5). Therefore, an ideal EV71 vaccine should be able to provide protection against most, if not all, EV71 genotypes.

Many approaches have been tested to develop a safe and effective EV71 vaccine (reviewed in [11], [12]). Among them, recombinant virus-like particles (VLPs) hold great promise, because of their advantages over inactivated whole-virion vaccine in terms of cost-effective production and safety (VLPs are absolutely non-infectious). Recombinant VLPs of the C2 genotype of EV71 have been shown to elicit the production of high-titer neutralizing antibodies in mice [13] and in macaque monkeys [14]. Passive immunization of maternal mice with EV71 VLPs confers protection to neonatal mice against lethal EV71 infection, demonstrating that neutralizing antibodies play an essential role in protection [13]. However, thus far, how the anti-VLP sera neutralize EV71 *in vitro* remains unclear.

In the present study, recombinant VLPs of the EV71 genotype C4 were produced using a baculovrius/insect cell expression system. The capability of the VLPs to induce neutralizing antibodies in mice was evaluated. Furthermore, mechanisms of EV71 neutralization by the anti-VLP sera were investigated.

## Materials and Methods

### Cells and Viruses

RD and Vero cells were grown as described previously [15]. EV71 strain G082 (genogroup C4) was propagated in RD cells. Virus titers were determined using RD cells by the microtitration method and expressed as the 50% tissue culture infective dose (TCID_50_) according to the Reed–Muench method [16]. Purified, inactivated EV71 was obtained from Hualan Inc. (Henan, China).

### Capsid Subunit Protein-specific Polyclonal Antibodies

The anti-VP0 guinea pig polyclonal antibody has been described previously [17]. The anti-VP3 guinea pig polyclonal antibody was generated by immunization of guinea pigs with the recombinant VP3 protein of coxsackievirus A16 (CVA16) [18], which was found to cross-react strongly with EV71 (data not show) and hence was used in this study to detect the VP3 protein of EV71. The anti-VP1 polyclonal antibody was generated by immunization of rabbits with recombinant EV71 VP1 protein produced from *Escherichia coli*.

### Vector Construction

RNA was extracted from RD cells infected with the EV71 strain G082 and subsequently reverse transcribed using oligo(dT) primers and M-MLV reverse transcriptase (Invitrogen, Carlsbad, CA, USA) according to the manufacturer's instructions. The resultant cDNA was used as a template for the amplification of gene fragments coding for P1 and 3CD, respectively. The P1 fragment was amplified with primers (forward 5′–AATCCATGGGTTCGCAGGTGTCT-3′ and reverse 5′- GACAAGCTTTCAAAGAGTAGTGATCGC-3′), digested with *Nco*I and *Hin*dIII, and then inserted into the plasmid pIExBac-1 (Novagen, Merck KGaA, Darmstadt, Germany) from the same sites, to make pIExBac-P1. The 3CD fragment was amplified with primers (forward 5′-AATCCATGGGCCCGAGCCTTGATTTT-3′ and reverse 5′-GACAAGCTTTCAAAATAACTCGAGCC-3′), digested with *Nco*I and *Hin*dIII, and inserted into pIExBac-1 from the same site, to make pIExBac-3CD.

### Generation of Recombinant Baculoviruses

pIExBac-P1 and pIExBac-3CD were used to generate the corresponding baculoviruses. The procedures, including vector transfection, and generation, selection and propagation of the recombinant viruses, were carried out according to the manufacturer's instructions (Novagen). For expression of proteins of interest, Sf9 insect cells were infected with recombinant baculoviruses. Infected cells and supernatants were harvested at 72 hours post-infection and subjected to biochemical analyses.

### ELISA and Western Blot Analyses of Protein Expression

Protein lysates from baculovirus-infected Sf9 cells were obtained by treating cells with a Tris–NaCl buffer containing 1% NP-40, followed by centrifugation at 12,000 rpm for 5 minutes. For indirect ELISA, wells of 96-well microtiter plates were coated with 5 µl lysate diluted in 50 µl PBS and incubated at 4 °C for 12 hours; then and after each of the following steps, the plates were washed three times with PBST buffer (1× PBS with 0.05% Tween 20). Consecutively, 200 µl/well of 5% milk in PBST was added for blocking and incubated at 37 °C for 1 hour; 50 µl/well of the primary antibodies (guinea pig anti-VP0 antiserum, rabbit anti-VP1 antiserum or guinea pig anti-VP3 antiserum) diluted 1:1000 in PBST plus 1% milk was added and incubated at 37 °C for 2 hours; then 50 µl/well of the corresponding horseradish peroxidase (HRP)-conjugated secondary antibodies (1:3000 diluted in PBST plus 1% milk) was added and incubated at 37 °C for 1 hour. For color development, 50 µl/well TMB mixture was added and incubated for 5–10 minutes; then 50 µl/well 1 N H_3_PO_4_ was added to stop the reaction. Absorbance was measured at 450 nm in a 96-well plate reader.

For Western blotting, protein samples were separated on 12% polyacrylamide gels and then transferred onto PVDF membranes. The membranes were probed with an antigen-specific primary antibody, followed by a corresponding HRP-conjugated secondary antibody. Positive signals on the membranes were developed by chemiluminescence using the BeyoECL Plus kit (Beyotime, Shanghai, China) and recorded using a LAS-4000 Luminescent Image Analyzer (Fujifilm Life Science USA, Stamford, CT, USA).

### Preparation of VLPs and Control Antigens for Immunization

Lysate from baculovirus-infected Sf9 cells was subjected to ultracentrifugation at 25,000 rpm for 6 hours on a 20% sucrose cushion. The resultant pellets were resuspended in PBS and then layered onto 10–50% sucrose gradients for ultracentrifugation at 39,000 rpm for 3 hours. Ten fractions were taken from top to bottom and assayed. Based on the SDS-PAGE and Western blotting results, VLP-rich fractions were pooled, dialyzed against PBS, and then pelleted by ultracentrifuge through a 20% sucrose cushion. The pelleted VLPs were resuspended in PBS and subsequently used for animal immunization. The VLP preparations were quantitated for VP0 content by Western blotting using the recombinant VP0 as a reference standard and anti-VP0 as the detection antibody. The uninfected Sf9 cell lysate was subjected to the same purification processes as the VLP, and served as the negative control antigen for immunization.

### Electron Microscopy

VLP samples were subjected to negative staining with 0.5% aqueous uranyl acetate, and transmission electron microscopy was performed with a Philips CM-12S microscope.

### Mouse Immunization

Prior to immunization, EV71 VLPs or similarly prepared negative control (Sf9) antigens were mixed with Imject alum (Pierce, Rockford, IL, USA) at a volumetric ratio of 1:1 according to manufacturer's instructions. Groups of five female Balb/c mice (6–8 weeks old) were injected intraperitoneally (i.p.) with the antigen/alum mixtures containing the VLPs (equivalent to 5 µg VP0) or the Sf9 lysate, respectively, at weeks 0, 2 and 5. Blood samples were collected before immunization and at week 7 when mice were terminated. Sera were kept at −80 °C until use. The animal studies were approved by the Institutional Animal Care and Use Committee at the Institut Pasteur of Shanghai.

### Antibody Measurement

To measure EV71-specific antibody responses in serum samples, 96-well ELISA plates were coated with inactivated EV71 (10 ng/well) and incubated for 3 hours at 37 °C. Plates were blocked with 5% milk in PBST for 1 hour at 37 °C. Subsequently, the plates were incubated with the serum samples diluted with 1% milk in PBST for 2 hours at 37 °C, and then with a HRP-conjugated anti-mouse IgG antibody (Sigma, St. Louis, MO, USA) for 1 hour at 37 °C. After color development, absorbance was determined at 450 nm in a 96-well plate reader. Endpoint titer was reported as the reciprocal of the highest serum dilution that had an absorbance ≥0.1 OD unit above the blank (absorbance of the preimmune samples).

### ELISPOT Assay

For B cell ELISPOT assay, spleens and bone marrow from three immunized mice were collected for each group at 3 weeks after the last immunization. The splenocytes and bone marrow cells were isolated, pooled, and counted. Ninety-six-well PVDF plates (Millipore, Billerica, MA, USA) were precoated with 100 ng/well of the purified inactivated EV71 and incubated overnight at 4 °C. Plates were blocked with complete RPMI 1640 medium for 2 hours at 37 °C. Freshly isolated spleen cells and bone marrow cells (1×10^5^/well) were added to the plates and incubated for 40 hours at 37 °C with 5% CO_2_. Subsequently, the plates were incubated with biotinylated goat anti-mouse IgG (0.1 µg/well) diluted in PBS buffer containing 1% FBS and 0.05% Tween-20 for 2 hours at 37 °C, and then with alkaline-phosphatase-conjugated streptavidin diluted 1:1000 in PBS for 1 hour at 37 °C. After washing the plate six times with water, 100 µl/well of NBT/BCIP substrate was added to the plates and incubated for 20 minutes for color development. The plates were washed with water to stop the reaction and then air dried. The antibody-secreting cell spots were imaged and counted using a CTL Immunospot reader (Cellular Technology Ltd., Shaker Heights, OH, USA).

### Neutralization Assay

Serum samples were diluted serially twofold using Dulbecco's Modified Eagle's Medium (DMEM) containing 2% FBS. The EV71 stock was diluted to a working concentration of 2 TCID_50_/µl. The neutralization assay was conducted using 96-well plates. In each well, 50 µl diluted antiserum was mixed with 50 µl EV71 containing 100 TCID_50_ and incubated for 1 hour at 37°C. Next, 100 µl cell suspension containing 15,000 RD cells was added to wells containing the virus/antiserum mixtures and incubated at 37°C with 5% CO_2_. After 3 days, the cells were observed to evaluate the appearance of cytopathic effects (CPEs). Neutralization titers were determined as the highest serum dilutions that could completely protect cells from CPE.

### Peptide ELISA

A series of 58 peptides spanning the entire amino acid sequence of VP1 of the EV71-G082 were synthesized by GL Biochem Ltd. (Shanghai, China). Each peptide consisted of 15 amino acid residues with 10 residues overlapping with the adjacent peptides. Wells of 96-well ELISA plates were coated with 400 ng of individual peptide in PBS buffer overnight at 4 °C. Then, the wells were blocked with 5% milk in PBST for 2 hours at 37 °C, incubated with the antiserum diluted in PBST plus 1% milk for 2 hours at 37 °C, followed by incubation with an HRP-conjugated secondary antibody for 1 hour at 37 °C. After color development, the absorbance was determined at 450 nm using a 96-well plate reader.

### Neutralization-inhibition Assay

Different concentrations of synthetic peptides were added to the anti-VLP sera diluted 1:5,000 in DMEM plus 2% FBS, and incubated for 1 hour at 37 °C. The peptide/antiserum mixtures were subjected to neutralization assay as described above. After 3 days, neutralization-inhibition was evaluated by analyzing cell viability using an MTT-based method. The medium in the 96-well plates was decanted gently. Then, 100 µl/well of 0.1%(w/v) 3-(4,5-dimethylthiazol-2-yl)-2,5-diphenyltetrazolium bromide (MTT) diluted in DMEM containing 2% FBS was added and incubated for 4 hours at 37 °C. The formazan crystals were solubilized with 100 µl DMSO. The absorbance was determined at 490 nm using a microtiter plate reader.

### Preattachment Assay

Antisera were diluted serially with DMEM plus 2% FBS. Then, 8 µl of the diluted antiserum or the control medium was added to 400 µl of the virus suspension containing 9×10^6^ TCID_50_ of EV71-G082, and incubated for 1 hour at 37 °C. The mixtures were then added to RD or Vero cells (3.5×10^5^ cells/well in 12-well plates) and incubated for 1 hour at 4 °C to allow virus attachment. After incubation, the cells were washed with cold PBS three times, collected, and subjected to Western blotting with anti-EV71 VP1 or anti-actin polyclonal antibodies.

### Postattachment Assay

EV71 virus stock was diluted with DMEM plus 2% FBS to a working concentration of 2 TCID_50_/µl. Then, 50 µl/well of the diluted virus or the control medium was added to 96-well microplates containing 1.5×10^6^/well of pre-seeded RD cells, and incubated for 1 hour at 4 °C to allow virus attachment. The cells were gently washed three times with DMEM to remove unbound virus. Subsequently, 50 µl/well of serially diluted antiserum was added and incubated with the cells for 1 hour at 37 °C. The cells were washed with DMEM three times before adding 100 µl DMEM. After 3 days, the cells were examined for CPE development.

## Results

### Expression and Assembly of Recombinant VLPs of EV71 Genotype C4

**Figure 1:**
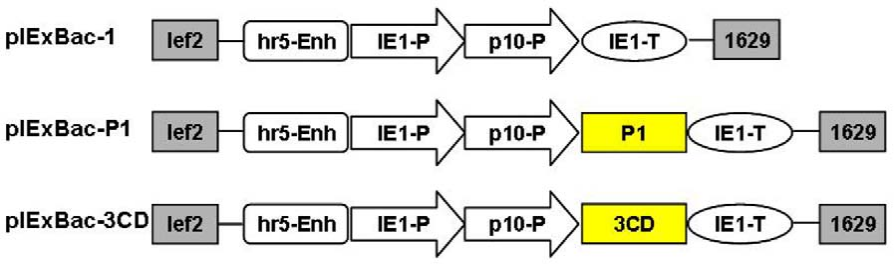
Diagrams of the constructs used in this study. lef2, the left sequence for homologous recombination; hr5-Enh, AcNPV hr5 enhancer; IE1-P, IE1 immediate early promoter; p10-P, p10 promoter; IE1-T, IE1 terminator; 1629, the right sequence for homologous recombination.

Two expression vectors, pIExBac-P1 and pIExBac-3CD (Figure 1), were constructed and subsequently used to generate the corresponding baculoviruses, namely IExBac-P1 and IExBac-3CD. Insect Sf9 cells were infected with the resultant recombinant baculoviruses separately or in combination.

**Figure 2:**
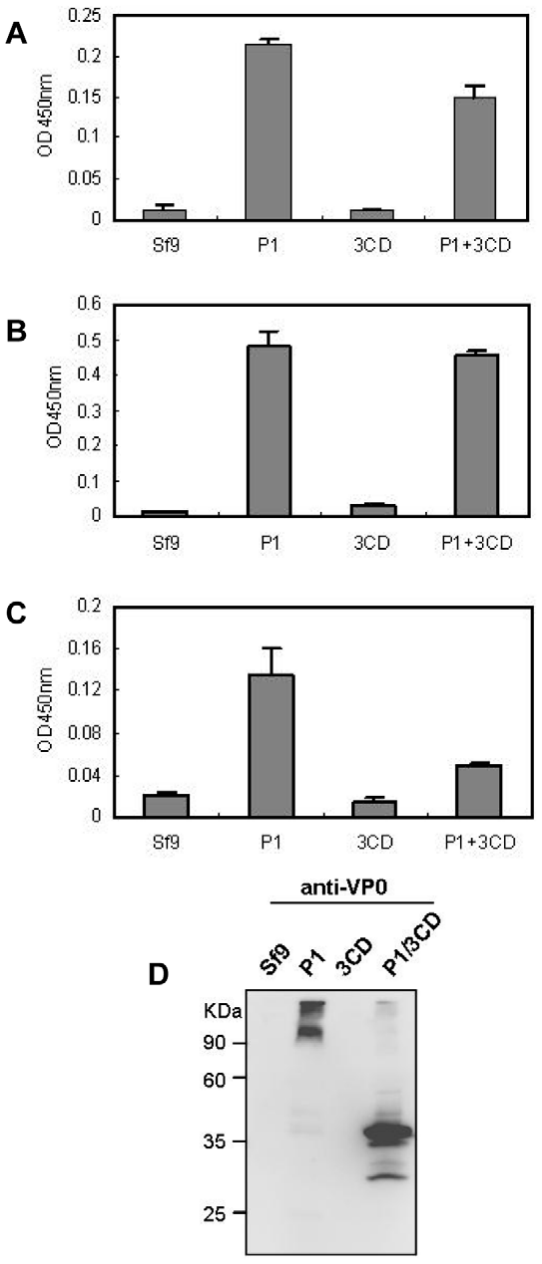
Coexpression of P1 and 3CD of EV71 in insect cells. Lysates from the baculovirus-infected Sf9 cells were analyzed by ELISA with (A) anti-VP0, (B) anti-VP1, or (C) anti-VP3 polyclonal antibodies, or (D) by Western blotting with the anti-VP0 polyclonal antibody. Sf9, mock-infected Sf9 cell lysate; P1, lysate from the IExBac-P1 infected cells; 3CD, lysate from the IExBac-3CD infected cells; P1+3CD, lysate from cells infected with both IExBac-P1 and IExBac-3CD. Error bars represent SE.

ELISA showed that the lysates from cells infected with the IExBac-P1 or co-infected with both the IExBac-P1 and IExBac-3CD reacted positively with the three polyclonal antibodies against VP0, VP1, or VP3, respectively; in contrast the IExBac-3CD infected cell lysate yielded only background level of readings for all three antibodies as did the mock-infected lysate (Figure 2A–C). The cell lysates were further analyzed by Western blotting with the anti-VP0 antibody to determine whether P1 could be processed. No signal was detected for the mock- or IExBac-3CD-infected samples (Figure 2D). For the IExBac-P1 lysate, two bands were evident:, one was around 100 kDa, probably representing the unprocessed full-length P1 polyprotein, and another appeared at the position of the loading well with a very high molecular weight, which could be aggregates due to the aberrant assembly of P1 (Figure 2D). The P1/3CD co-infected sample produced a major band of ∼38 kDa and a minor band of ∼28 kDa (Figure 2D), which are expected for processed VP0 and VP2 subunit proteins. Taken together, these results indicate that only the IExBac-P1/IExBac-3CD combination resulted in efficient production and correct processing of the structural protein P1, which is the pre-requirement for EV71 particle assembly. Therefore, only the IExBac-P1/IExBac-3CD combination was used for subsequent studies.

**Figure 3:**
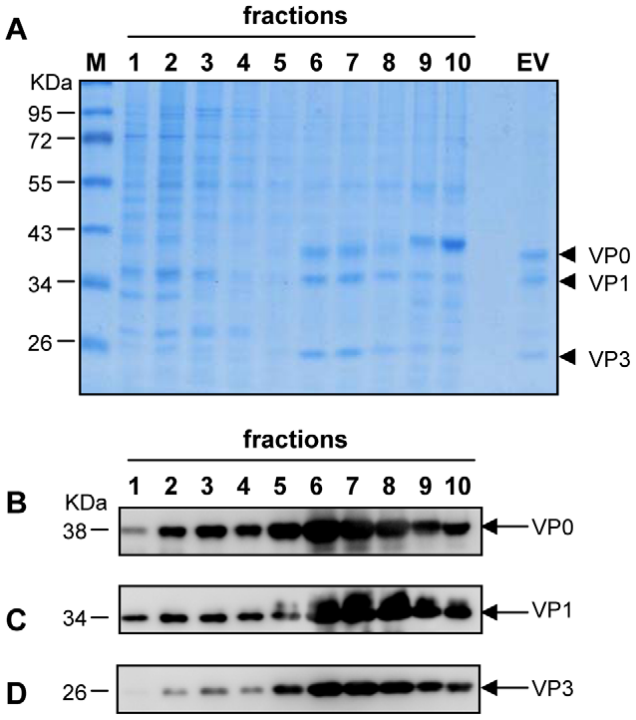
Sucrose gradient analysis. Lysates were layered onto 10–50% sucrose gradients for ultracentrifugation. Ten fractions were taken from top to bottom. (A) SDS-PAGE analysis. The ten fractions were separated by SDS-PAGE, and subsequently stained with Coomassie Blue dye. M, protein marker. EV, purified whole virion EV71 standard from Hualan Inc. (B) Western blotting with anti-VP0 polyclonal antibody. (C) Western blotting with anti-VP1 polyclonal antibody. (D) Western blotting with anti-VP3 polyclonal antibody.

**Figure 4:**
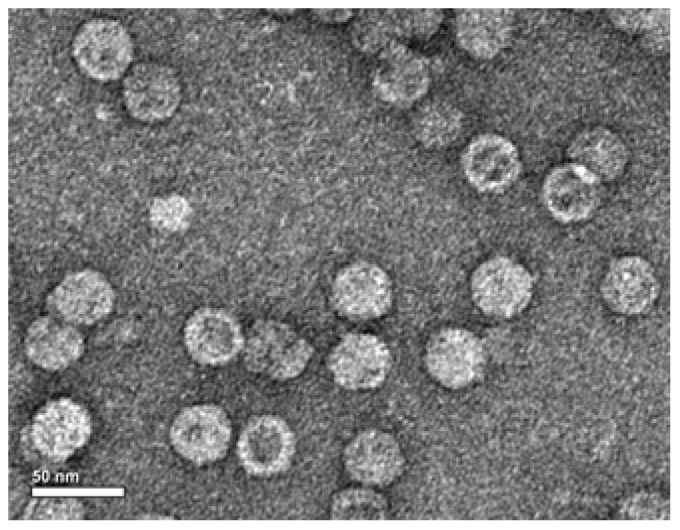
Electron microscopy of EV71 VLPs. Partially purified VLPs were negatively stained with 0.5% aqueous uranyl acetate and visualized by transmission electron microscopy. Bar = 50 nm.

To evaluate VLP assembly, the P1/3CD co-infected cell lysate was subjected to sucrose gradient sedimentation. SDS-PAGE of the resultant gradient fractions showed that three major bands of 38, 34 and 26 kDa were evident for fractions #6 and #7, and a purified authentic EV71 standard (Figure 3A). Western blotting with three capsid subunit protein-specific antibodies revealed that the 38, 34 and 26-kDa bands represented VP0, VP1 and VP3, respectively (Figure 3B–D). These data strongly suggest that processed VP0, VP1 and VP3 co-assembled into particles. Electron microscopy of fractions #6 and #7 confirmed the presence of VLPs with a diameter of ∼30 nm (Figure 4).

### VLP Immunization Elicited EV71-specific Antibody Responses in Mice

**Figure 5:**
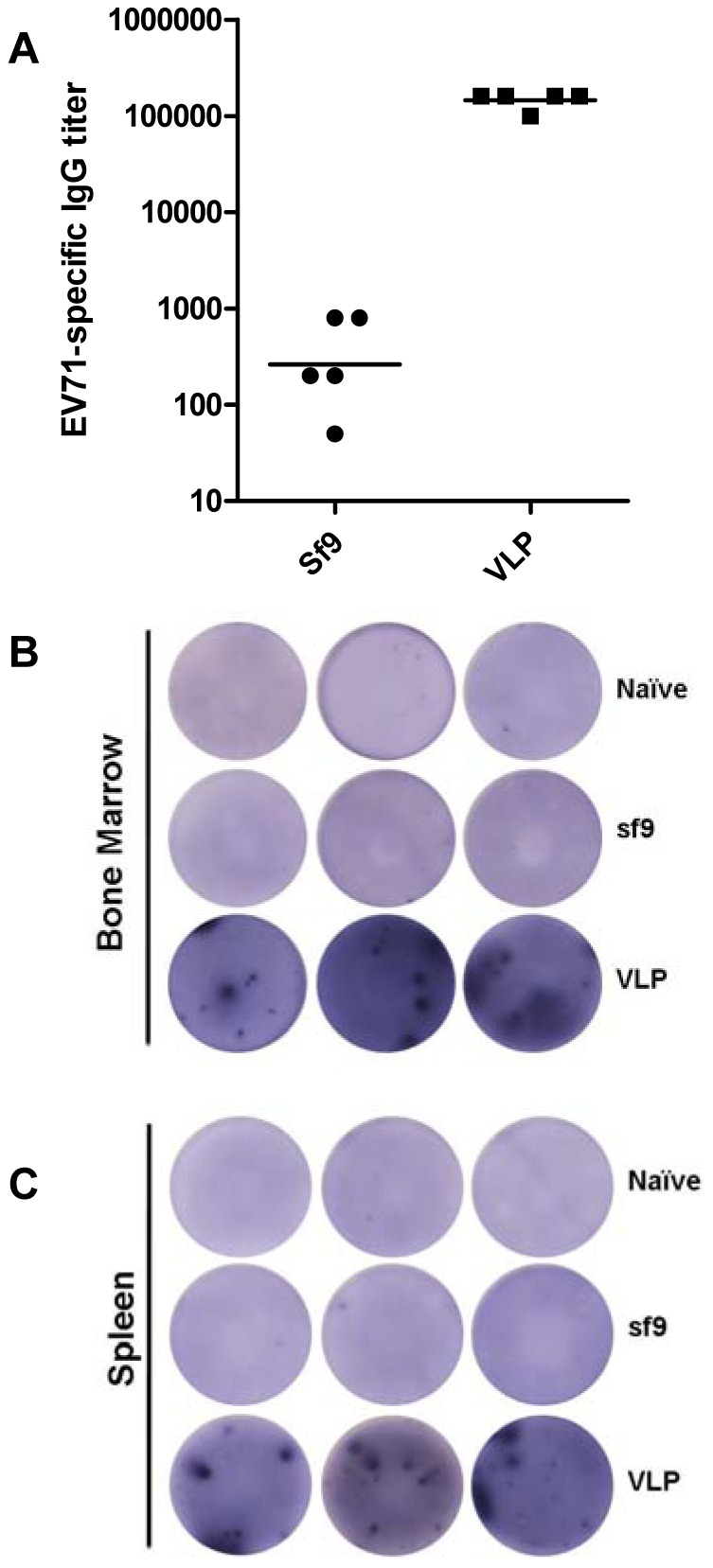
Antibody responses following VLP immunization. Groups of five mice were injected i.p. at weeks 0, 2 and 5, with alum-absorbed antigens: EV71-VLP equivalent to 5 µg VP0, or the control lysate similarly prepared from uninfected Sf9 cells. The immunized mice were sacrificed at week 7 (2 weeks after the last immunization), and the serum, bone marrow and spleen samples were collected and assayed. Results are representative of two independent experiments. (A) EV71-specific serum IgG titers of the Sf9 and the VLP groups. Each symbol represents a mouse, and the line indicates the geometric mean value of the group. (B) ELISPOT analysis of EV71-specific antibody-secreting cells in mouse bone marrow. Samples from naïve mice were used as the control. Results of triplicate wells are shown. (C) ELISPOT analysis of EV71-specific antibody-secreting cells in mouse spleens. Samples from naïve mice were used as the control. Results of triplicate wells are shown.

Mice were immunized i.p. with partially purified EV71 VLP or the control antigen similarly prepared from mock-infected Sf9 lysates. After three doses, all VLP-immunized mice generated serum antibodies that reacted with inactivated EV71 at high titers; in contrast, the Sf9 lysate-immunized mouse sera had only background levels of reactivity (Figure 5A). ELISPOT assay further demonstrated the presence of EV71-specific antibody-secreting cells in both bone marrow and spleens of the VLP-vaccinated mice, but not of the Sf9 lysate-immunized or naïve mice (Figure 5B and C).

### Neutralization Capacity of VLP-immunized Sera

**Figure 6:**
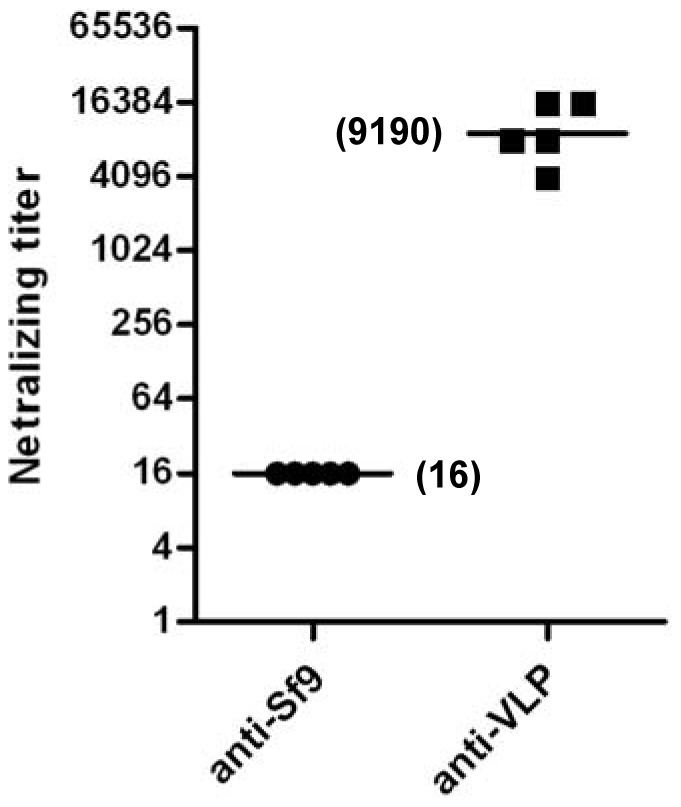
VLP-immunized sera efficiently neutralized EV71 infection *in vitro*. Neutralization titers of the antisera were determined by TCID_50_ reduction assay. The antisera of the Sf9 group did not show any neutralization at 1:32 (the lowest dilution tested) and were therefore assigned a titer of 16 for GMT computation. Each symbol represents a mouse, and the line indicates the GMT of the group. Results are representative of two independent experiments.

Sera from the VLP- or Sf9 lysate-immunized mice were evaluated for their capacity to neutralize live EV71 *in vitro*. The anti-Sf9 sera did not exhibit any neutralization effect at 1:32 (the lowest dilution tested) and were therefore assigned a titer of 16 for geometric mean titer (GMT) computation; in contrast, all VLP-immunized sera potently inhibited the infection, with a neutralizing GMT of 9190 (Figure 6).

### Neutralizing Antibodies in Anti-VLP Sera Predominantly Target an Epitope Located in the GH Loop of VP1 Protein

**Figure 7:**
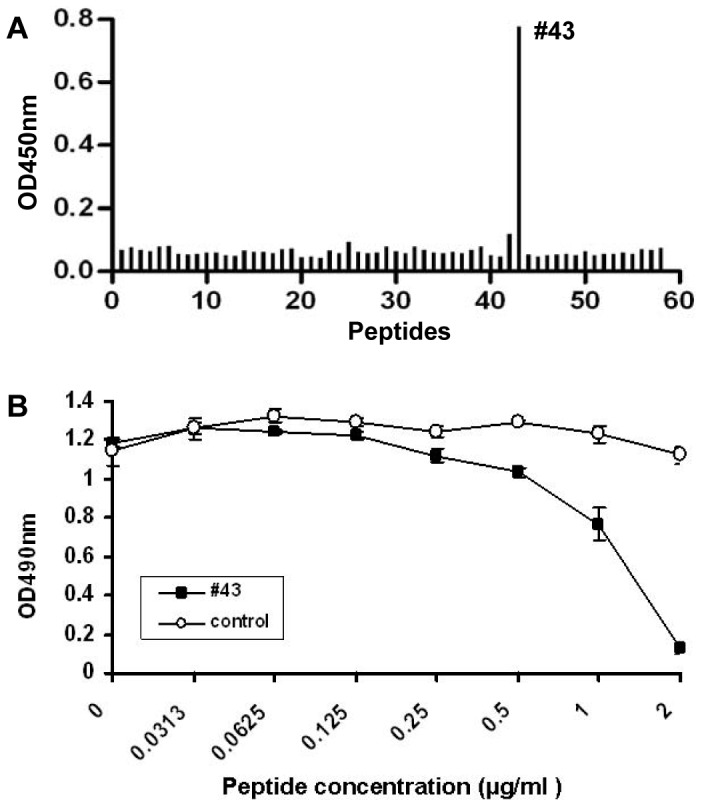
Neutralizing antibodies in the anti-VLP sera predominantly target an epitope located in the GH loop of VP1 protein. (A) Mapping of immunodominant B-cell linear epitopes within the VP1 protein. The pooled anti-VLP sera were analyzed by indirect ELISA for reactivity with a panel of 58 synthetic peptides covering the entire sequences of VP1. (B) Neutralization-inhibition by #43 peptide. The anti-VLP sera were incubated with #43 peptide or a negative control peptide (from HIV) at different concentrations for 1 hour at 37 °C. The peptide/antiserum mixtures were subjected to neutralization testing. Neutralization-inhibition by the peptides was determined using an MTT method. Data are means±SD of the OD490 readings of triplicate wells.

The anti-VLP sera were tested by ELISA for reactivity with a panel of 58 synthetic peptides covering the entire VP1 region. As shown in Figure 7A, the anti-VLP sera reacted predominantly with the #43 peptide (FGEHKQEKDLEYGAC), which overlaps with the SP70 epitope (YPTFGEHKQEKDLEY) identified previously [19]. According to the crystal structure of EV71 [20], [21], the #43 peptide was located in the GH loop of VP1 protein. These results indicate that a dominant linear epitope lies within this GH loop. To determine the contribution of this epitope in generation of neutralizing antibodies, the anti-VLP sera were incubated with different concentration of the #43 peptide prior to neutralization tests. Figure 7B shows that the #43 peptide decreased the neutralization capacity of the anti-VLP sera in a dose-dependent manner, while the control HIV peptide did not interfere with neutralization. These results indicate that the neutralizing antibodies predominantly target the epitope within the #43 peptide sequence.

### Anti-VLP Sera Blocked Virus Attachment to Cells

**Figure 8:**
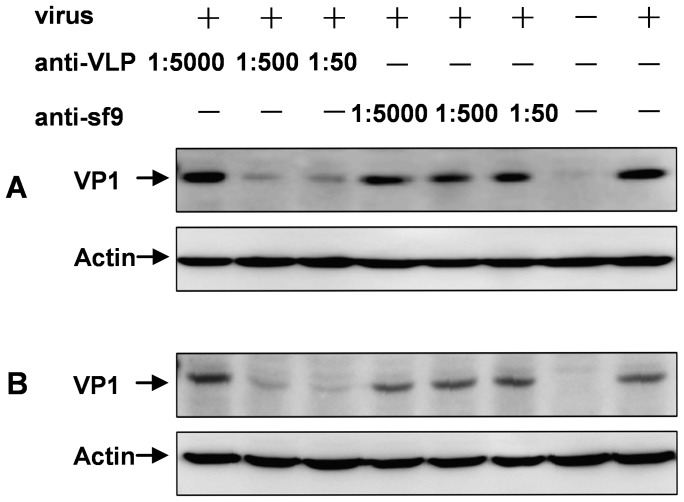
Inhibitory effect of the antisera on EV71 attachment to cells. Antisera at different dilutions (1:50, 1:500, and 1:5,000) were mixed with 400 µl of EV71 containing 9×10^6^ TCID_50_, and incubated for 1 hour at 37 °C. The mixtures were added to (A) RD or (B) Vero cells for incubation for 1 hour at 4 °C to allow virus attachment to the cells. After incubation, the cells were washed with cold PBS three times, collected, and subjected to Western blotting with a polyclonal antibody against EV71 VP1. For loading control, an antibody against actin was also used in Western blotting.

To investigate mechanisms of EV71 neutralization, we first tested whether preincubation with antisera could prevent the virus from binding to cells. Figure 8 shows that preincubation with the anti-Sf9 sera did not affect EV71 attachment, regardless of antiserum dilution; in contrast, treatment with the anti-VLP significantly reduced virus binding to RD (Figure 8A) or Vero (Figure 8B) cells in a dose-dependent manner.

### Postattachment Treatment with Anti-VLP Sera Inhibited *In Vitro* Infection

**Figure 9:**
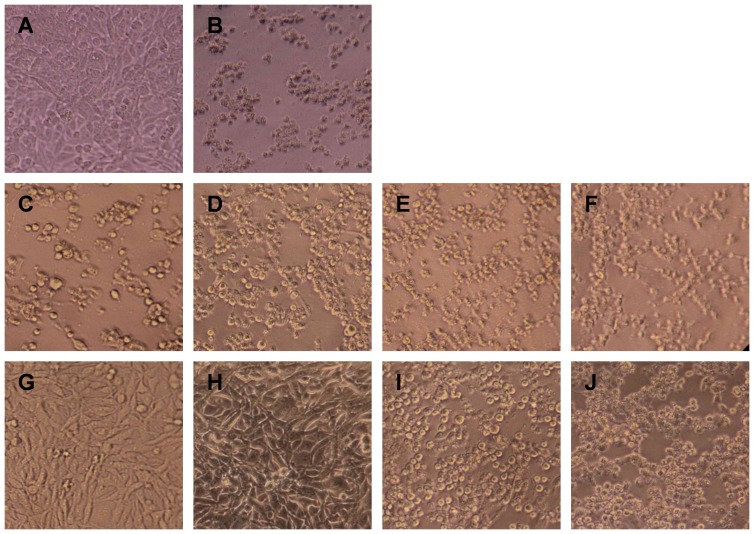
Postattachment neutralization by the anti-VLP sera. EV71 was allowed to attach to RD cells by incubation for 1 hour at 4 °C. The virus-attached cells were then treated for 1 hour at 37 °C with the antisera at different dilutions as indicated. Three days later, the treated cells were observed for CPE. (A) uninfected cells. (B) EV71-bound cells without antisera treatment. (C–F) EV71-bound cells were treated with the anti-Sf9 diluted 1:250, 1;500, 1:1000, and 1:2000, respectively. (G–J) EV71-bound cells were treated with the anti-VLP diluted 1:250, 1;500, 1:1000, and 1:2000, respectively.

To determine whether the antisera could neutralize infection at a post-attachment step, the virus was allowed to bind target cells by incubation at 4 °C for 1 hour. Virus-attached cells were then treated with serially diluted antisera. Three days later, the cells were observed for the appearance of CPE. As expected, the anti-Sf9 did not provide protection regardless of the dilution factors (Figure 9C–F). Surprisingly, treatment with the anti-VLP sera at 1:250 or 1:500 dilutions prevented cells from developing a CPE, whereas the 1:1,000 and 1:2,000 diluted antisera failed to do this (Figure 9G–J). These results indicated that the anti-VLP sera were able to inhibit infection at a postattachment stage; however, neutralization at this stage required much higher concentration of neutralizing antibodies than that at the preattachment stage.

## Discussion

The present study demonstrates the successful production of recombinant VLPs of EV71 genotype C4 and their potency in eliciting high-titer neutralizing antibodies. The antisera against VLPs of EV71 (C4 genotype) were able to neutralize efficiently a panel of six C4 strains isolated from different locations in China and in different years (data not shown). Whether the anti-VLP sera can cross-neutralize other EV71 genotypes remains to be determined. Previously, Chung et al. have shown that VLPs of EV71 genotype C2 could be generated [13], [22], and they could induce the production of neutralizing antibodies to protect mice against lethal challenge [13]. Together, these data indicate that it is feasible to produce VLPs of different genotypes and subsequently combine them to formulate a multivalent EV71 VLP vaccine. Recently, our group also demonstrated the production and vaccine potential of recombinant VLPs of CVA16 [23], which is another major causative agent of HFMD. Therefore, it is possible to develop a combined vaccine consisting of both EV71 and CVA16 VLPs for broad protection against HFMD. Success of this kind of multivalent VLP vaccine strategy has been demonstrated by the commercialization of bi- and tetravalent HPV vaccines.

The present study showed that preincubation of EV71 with the anti-VLP neutralizing antibodies inhibited virus attachment to the permissive cells. Surprisingly, we also found that treating the virus-bound cells with the anti-VLP sera could neutralize infection. It is likely that antiserum binding interferes with the virus uncoating process after initial attachment, probably by arresting capsid conformational change, which is required for uncoating to release infectious RNA genome into the cells. However, whether or not this is true remains to be determined.

In the present study, we attempted to map for the potential linear neutralizing epitopes within VP1 by using a panel of 58 synthetic peptides spanning the entire VP1 region. We chose VP1 as the target for mapping the neutralizing epitopes, as recombinant VP1, but not VP0 or VP3, has been shown to induce the production of neutralizing antibodies [24], [25], [26]. We found that the neutralizing antibodies in the anti-VLP mouse sera mainly bound the #43 peptide (FGEHKQEKDLEYGAC), and pretreatment with this peptide significantly decreased the neutralization capacity of the anti-VLP sera, indicating that the #43 peptide contains a dominant neutralizing linear epitope, which is in agreement with two previous reports [19], [27]. The mechanism of neutralization by the antibodies targeting the #43 peptide/epitope has not been revealed. The #43 peptide is located at the GH loop of VP1 and is exposed on the capsid surface according to the crystal structure of EV71 [20], [21]. It is thus likely that the domain containing this peptide/epitope is involved in virus attachment to permissive cells. Therefore the corresponding neutralizing antibodies may block cell attachment. In addition, it was proposed by Wang *et al.* [20] that the GH loop of VP1 acts as an adaptor-sensor for the EV71 uncoating process after cell attachment. Therefore, antibodies against this epitope may also be able to shield the sensor and, by doing so, block virus uncoating and the downstream post-attachment processes.

The #43 peptide/epitope is completely conserved among EV71 genogroups [19]. Thus, it is rational to believe that EV71 VLPs can elicit neutralizing antibodies against a broad spectrum of EV71 clinical strains. Although the immunogenicity of the VLPs in mouse studies may not be fully translated into their immunogenicity in humans, the results from this study and others provide a solid foundation for the further development of VLP-based human vaccines against EV71 and other enteroviruses.

In conclusion, we demonstrated that the VLPs of EV71 subgenogroup C4 can be recombinantly produced and can elicit the production of neutralizing antibodies in mice. We further showed that the neutralizing anti-VLP sera were able to inhibit *in vitro* EV71 infection at both pre- and postattachment steps. These findings represent a valuable addition to the understanding of mechanisms of EV71 neutralization, and have strong implications in the development of VLP-based vaccines against infection with EV71 and/or other enteroviruses.
